# EGCG Regulates the Effect of HDAC6 on Oxidative Stress of Human Periodontal Ligament Fibroblasts Induced by Lipopolysaccharide

**DOI:** 10.1002/iid3.70198

**Published:** 2025-04-27

**Authors:** Yang Jie, Liu Xia, Peng ZeHui, Long YuanZhu, Chen Bin, Li Yaohua, Gao Si, Bai GuoHui, Liu JianGuo, Fan Qin

**Affiliations:** ^1^ Affiliated Stomatological Hospital Zunyi Medical University Zunyi China; ^2^ Kweichow Moutai Hospital Zunyi China; ^3^ Key Laboratory of Oral Disease Research, School of Stomatology Zunyi Medical University Zunyi China

**Keywords:** epigallocatechin gallate, HDAC6, Keap1/Nrf2/HO‐1, periodontitis

## Abstract

**Background:**

Epigallocatechin gallate (EGCG) has anti‐inflammatory and antioxidative stress effects in periodontitis. However, the specific mechanisms involved remain unclear. Our study explored whether the mechanism by which EGCG on alleviates inflammation and oxidative stress in human periodontal ligament fibroblasts (hPDLCs) involves HDAC6.

**Methods:**

We treated hPDLCs with lipopolysaccharide (LPS) and EGCG, and detected the resultant effects on cell proliferation by the CCK‐8 method. Cells were divided into three groups: control, LPS, and EGCG + LPS. The expression of tumor necrosis factor α (TNF‐α) and interleukin‐1β (IL‐1β) was detected by enzyme‐linked immunosorbent assay (ELISA), and the expression of reactive oxygen species (ROS) was detected using 2′,7′‐dichlorofluorescein diacetate. The expression of histone deacetylase 6 (HDAC6), p62, heat shock protein 70 (Hsp70), Kelch‐like ECH‐associating protein (Keap1), nuclear factor E2‐related factor 2 (Nrf2), and heme oxygenase‐1(HO‐1) mRNA was detected by real‐time quantitative polymerase chain reaction (RT‐qPCR). The protein expression of HDAC6, Nrf2, and nod‐like receptor protein 3 (NLRP3) was detected by western blotting.

**Results:**

At concentrations of less than 100 μmol/L, EGCG can promote cell proliferation and significantly inhibit the levels of TNF‐α and IL‐1β. Moreover, EGCG can activate the Nrf2 pathway and inhibit ROS production. Furthermore, EGCG inhibited the expression of HDAC6 and promoted the expression of p62 and Hsp70, indicating that the anti‐inflammatory and antioxidant effects of EGCG are closely related to HDAC6.

**Conclusions:**

EGCG can regulate LPS‐induced oxidative stress levels of hPDLCs through the Keap1/Nrf2/HO‐1 pathway and reduce the expression of HDAC6‐related factors. Therefore, HDAC6 may be a potential target for EGCG in the treatment of periodontal inflammation and oxidative stress.

## Graphical abstract

(1) At concentrations of less than 100 μmol/L, EGCG can promote cell proliferation and significantly inhibit the levels of TNF‐α and IL‐1β. (2) EGCG can activate the Nrf2 pathway and inhibit ROS production. (3) EGCG inhibited the expression of HDAC6 and promoted the expression of p62 and Hsp70, indicating that the anti‐inflammatory and antioxidant effects of EGCG are closely related to HDAC6.

## 1 Introduction

Periodontitis is an important cause of intraoral tooth loss and its prevalence rate is high. The existing periodontal treatment methods are limited, mainly periodontal initial therapy, supplemented by drug therapy; However, the traditional subgingival scaling can cause a lot of traumas to the periodontal, and there are few options for drug treatment, necessitating the urgent development of relevant therapeutic strategies. In response to stimulation by periodontal pathogens, the nuclear factor‐κB (NF‐κB) inflammatory pathway is activated in periodontal tissue to produce pro‐inflammatory factors that lead to periodontal inflammation. Simultaneously, immune cells in the periodontium produce reactive oxygen species (ROS) through the respiratory burst mechanism, which promotes the antioxidative stress response [1, 2]. However, with the accumulation of ROS, and the aggravation of tissue inflammation, tissue destruction occurs as a result of both inflammatory and oxidative stress reactions. In recent years, studies have found that immunodeficiency, diabetes, smoking, and stress may induce epigenetic modifications and destroy the balance between the host and microorganisms, promoting the occurrence and development of periodontitis. Therefore, certain individuals are at higher risk for severe periodontitis [3]. Histone deacetylase 6 (HDAC6) is an important protein in epigenetic modification and is closely related to the pathogenesis of inflammatory diseases. It promotes the transformation of interleukin‐1β (IL‐1β) and caspase‐1 by participating in the assembly of the nod‐like receptor protein 3 (NLRP3) microtubule center of inflammatory bodies [4]. Studies have shown that HDAC6 inhibition can alleviate neurodegenerative diseases [5]. Overexpression of HDAC6 suppress the acetylation of p62, thereby inhibiting the antioxidant effect of Keap1/Nrf2/HO‐1 pathway [6]. Increasing evidence shows that HDAC6 is involved in hypoxia‐induced gene transcription regulation and the pathogenesis of various diseases. Studies have found that HDAC6 inhibitors can activate Nrf2 and play a neuroprotective role in Alzheimer's disease, Parkinson's disease and others [7]. Therefore, HDAC6 may be a potential target in the treatment of periodontitis.

Epigallocatechin gallate (EGCG), the most abundant catechin in tea polyphenols, is a flavonoid‐3‐ethanolphenol compound with eight free hydroxyl groups [8]. EGCG is an effective antioxidant that can play anti‐inflammatory roles and reduce antioxidative stress in periodontitis [9]. By comparing the effect of EGCG aqueous solution and distilled water in a new‐type ultrasonic scaler tip, it has been found that the new‐type scaler tip using EGCG aqueous solution can increase the clinical attachment level of patients’ periodontal; [10] however, the exact underlying mechanism remains unclear. Some studies have shown that EGCG can inhibit the expression of HDAC6 through an epigenetic pathway, thus exerting anticancer effects on cells [11]. In this study, we established an inflammatory model of human periodontal ligament fibroblasts (hPDLCs) to verify the anti‐inflammatory and antioxidant effects of EGCG. Furthermore, we performed western blot and real‐time quantitative polymerase chain reaction (RT‐qPCR) to detect the effect of EGCG on the expression of HDAC6‐related factors, so as to explore the mechanism underlying the role of EGCG in the prevention and treatment of periodontitis. The findings of this study provide valuable reference and theoretical support for the development of effective clinical therapies against periodontitis.

## 2 Materials and Methods

### 2.1 Cell Isolation and Culture

The present study was approved by the Ethics Committee of the Affiliated Stomatological Hospital of Zunyi Medical University (opinion No.: YJSKTLS‐2020‐2023‐012H). Primary periodontal ligaments were obtained from human third molars and premolars that were extracted for orthodontic reasons. Briefly, the periodontal ligament tissues attached to the mid‐third of the root surfaces were scraped off with a sharp surgical scalpel, followed by treatment with 2 mg/mL Type I collagenase (Solarbio, China) for 40 min. Then, the tissues were cultured in dishes with growth medium at 37°C, in air with 95% humidity plus 5% CO_2_, and the medium was changed every 3 days. The growth medium consisted of 89% DMEM (Livning, China), 10% fetal bovine serum (Livning, China), and 1% penicillin‐streptomycin (Solarbio, China). When the cells reached 80%–85% confluence, they were digested with 0.25% trypsin (Solarbio, China) for further passaging. Passages 3–5 were used in the present study.

### 2.2 Cell Proliferation

hPDLCs were inoculated into a 96‐well plate, with 1 × 10^4^ cells per well, and cultured for 24 h. Subsequently, cells were treated with medium containing *Escherichia. coli* LPS (Sigma, America) at the dose of 0, 5, 10, and 20 μg/mL for 24 h. Similarly, cells were treated with medium containing EGCG (0, 5, 10, 20, 50, 100 μmol/L) for 6, 12, and 24 h. Then, 10 μL of CCK‐8 reagent was added to each well and the cells were treated with LPS and EGCG. The absorbance value was recorded by a Thermo Scientific microplate absorbance reader at 450 nm (Thermo Scientific, America).

### 2.3 Enzyme‐Linked Immunosorbent Assay (ELISA)

ELISA kit (Elabscience, China) was used to detect the expression levels of tumor necrosis factor α (TNF‐α) and IL‐1β. hPDLCs were inoculated into a 6‐well plate, with 2 × 10^5^ cells per well, and cultured for 24 h. LPS and EGCG were diluted in a medium containing serum. After cells was cultured in the medium containing LPS (0, 5, 10, 20 μg/mL) for 24 h, or pretreated EGCG (0, 5, 10, 20, 50, 100 μmol/L) for 2 h before LPS for 24 h, the supernatant of the cells was collected after centrifugation. The supernatant will be operated according to the manufacturer's instructions (Elabscience, China). The OD value of each well was detected by 450 nm wavelength on the Thermo Scientific microplate absorbance reader, and the expression of TNF‐α and IL‐1β in each group was calculated.

### 2.4 Quantification of ROS

ROS levels were detected using 2′,7′‐dichlorofluorescein diacetate (DCFH‐DA) (Beyotime, China). hPDLCs were inoculated into a culture plate with 1 × 10^6^ cells. The cells were cultured in medium containing EGCG (0, 5, 10, 20, 50, 100 μmol/L) for 2 h, then LPS (5 μg/mL) was added to each group, and no drugs were added to the control group. The cells were cultured for 24 h. After drug treatment, the digested cells were transferred to the centrifuge tube, DCFH‐DA was added, incubated for 20 min, and then the cells were washed with serum‐free medium. The treated cells were placed in a fluorescence enzyme labeling instrument, with excitation wavelength of 488 nm and emission wavelength of 525 nm for detection.

### 2.5 Real‐Time Quantitative Polymerase Chain Reaction

RT‐qPCR was used to detect the relative changes in the mRNA expression levels of nuclear factor E2‐related factor 2 (Nrf2), Kelch‐like ECH‐associating protein (Keap1), heme oxygenase‐1 (HO‐1), p62, heat shock protein 70 (Hsp70), and HDAC6. hPDLCs were inoculated into a 6‐well plate, with 2 × 10^5^ cells per well. Cells were treated with the 100 μmol/L EGCG for 2 h, and then co‐cultured with LPS (5 μg/mL) for 24 h. Cells were lysed using Trizol Reagent (TaKaRa, Tokyo, Japan) to extract the total mRNA and then converted to synthesize cDNA using a Primer Script RT Reagent Kit (TaKaRa, Tokyo, Japan). qPCR was performed according to the instructions of TB Green Premix Ex TAQTM‐II PCR amplification kit (TaKaRa, Tokyo, Japan), and the expression level of related mRNA was detected. GAPDH was used as an internal reference. The primer sequence was shown in Table 1.

**Table 1:** Primer sequence.

Gene	Primer sequence (5′ → 3′)
Nrf2 F	AGTCCAGAAGCCAAACTGACAGAAG
Nrf2 R	GGAGAGGATGCTGCTGAAGGAATC
HO‐1 F	TGCCAGTGCCACCAAGTTCAAG
HO‐1 R	TGTTGAGCAGGAACGCAGTCTTG
Keap1 F	TGAGCCAGAGCGGGATGAGTG
Keap1 R	CGGCATAAAGGAGACGATTGAGGAC
p62 F	TGATTGAGTCCCTCTCCCAGATGC
p62 R	CCGCTCCGATGTCATAGTTCTTGG
Hsp70 F	CAGTGGAGATAGTTGGTGGTGCTAC
Hsp70 R	AAGCAGGCGATAAGATGGCACAC
HDAC6 F	AATGTAGAGGAGAGCGAGGAGGAAG
HDAC6 R	AGACCAGCCCTGTGCGAGAC
GAPDH F	CAGGAGGCATTGCTGATGAT
GAPDH R	GAAGGCTGGGGCTCATTT

Abbreviations: F = forward, R = reverse.

### 2.6 Western Blot

Western blot was used to detect the expression of related proteins. hPDLCs were inoculated into a T25 culture flask with 1 × 10^6^ cells, and cultured for 24 h. EGCG and LPS‐treated cells in the same way as RT‐qPCR. The cells were lysed with RIPA reagent (Elabscience, China) for protein extraction and a BCA kit (Solarbio, China) was used for protein detection; then, the, protein sample (loading amount 30 μg) and marker (3 μL) were subjected to 10%–12% SDS‐PAGE. Next, the sample was transferred to a PVDF membrane, followed by incubation with the primary antibody at 4°C overnight. After incubation with the secondary antibody, the reactivity bands were visualized using the ECL kit.

### 2.7 Statistical Analysis

The statistical software SPSS 18.0 was used to analyze all the results, and the measurement data were represented by mean ± standard deviation $\left(\bar{x}\pm s\right)$. Multiple comparisons were made between different groups using *t*‐test and one‐way analysis. *p* < 0.05 indicated that the difference was statistically significant.

## 3 Results

### 3.1 Cell Viability

**Figure 1:**
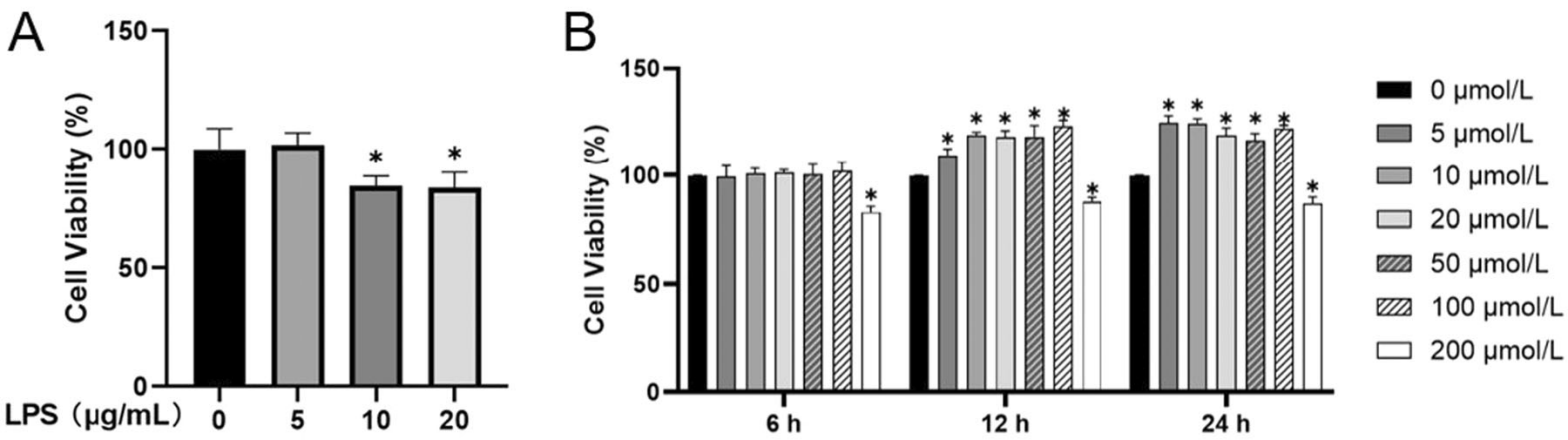
At a concentration of 5 μg/mL, LPS had no inhibitory effect on the cell viability of hPDLCs; however, at 10 and 20 μg/mL, LPS significantly inhibited the cell viability of hPDLCs (A). At 5–100 μmol/L, EGCG promoted the proliferation of hPDLCs at 12 and 24 h, and there was no significant difference in cell viability between 12 and 24 h (B).

The effects of LPS and EGCG on the proliferation of hPDLCs were detected by CCK‐8 assay. At the concentration of 5 μg/mL, LPS had no significant inhibitory effect on cell proliferation; however, at concentrations of > 5 μg/mL, LPS could significantly inhibit cell viability and proliferation (Figure 1A). Treatment with EGCG at the concentration of 5–100 μmol/L for 6, 12, and 24 h had no inhibitory effect on the proliferation of hPDLCs; however, the proliferation of the EGCG group was higher than that of the control group within 12 and 24 h. In addition, when the EGCG concentration was 200 μmol/L, cell viability at 6, 12, and 24 h was lower than that in the control group (Figure 1B).

### 3.2 Effects of EGCG on TNF‐α, IL‐1β, and ROS in hPDLCs

**Figure 2:**
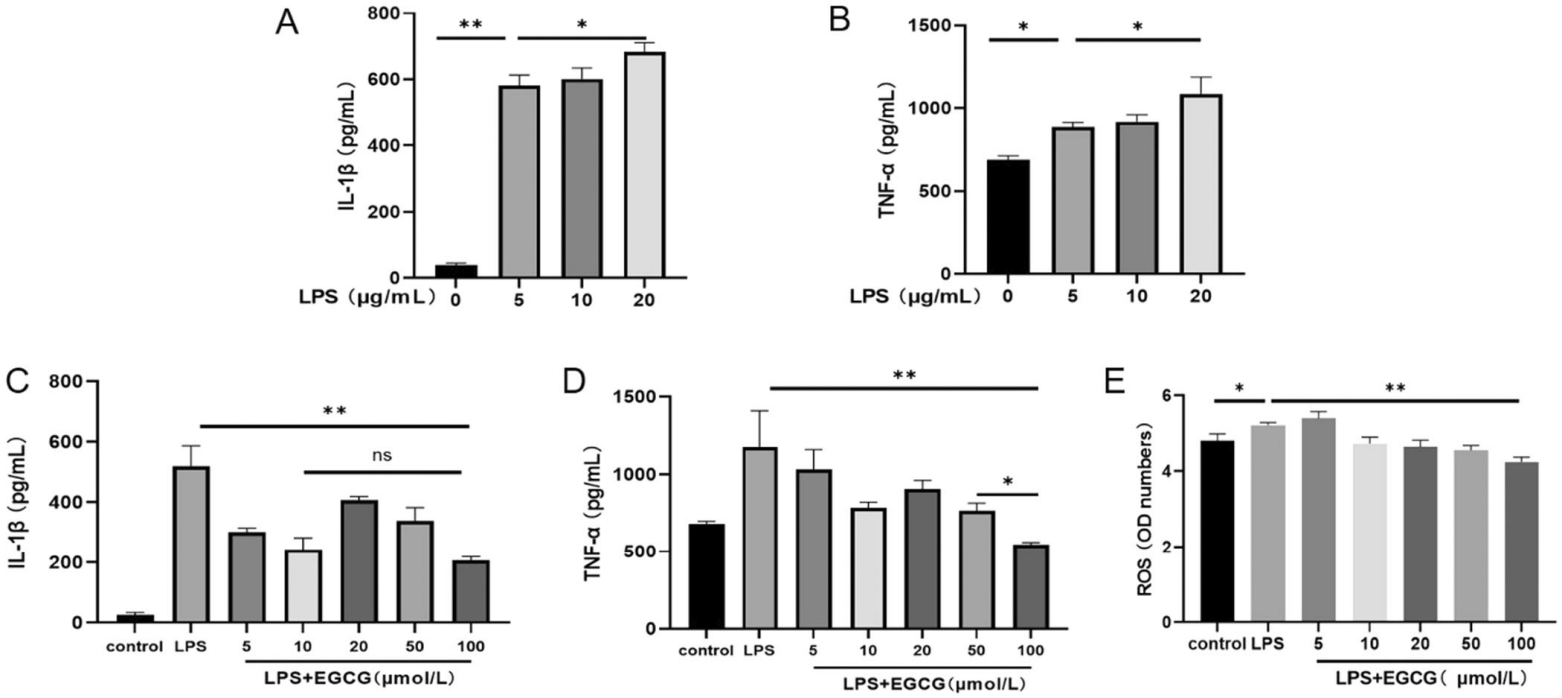
The expression of IL‐1β and TNF‐α increased after 24 h of hPDLCs treated by LPS (A and B). Compared with the LPS (5 μg/mL) group, the expression of IL‐1β and TNF‐α was significantly inhibited after the addition of EGCG, and the inhibitory effect was most obvious when the EGCG concentration was 100 μmol/L (C and D). When EGCG concentration was 100 μmol/L, LPS‐induced ROS expression was significantly inhibited (E). **p < 0.01, *p < 0.05, ns, not significant.

Following the treatment of hPDLCs with LPS, the expression of TNF‐α and IL‐1β was higher than in the control group, in a concentration‐dependent manner (Figure 2A,B). Based on the results for cell viability, 5 μg/mL was used as the dosage of LPS in the subsequent experiment. To verify the effect of EGCG on inflammation in hPDLCs, the secretion of TNF‐α and IL‐1β in each group was detected by ELISA. The results showed that the expression of TNF‐α and IL‐1β in the LPS group was higher than in the control group and EGCG group; furthermore, EGCG inhibited the secretion of TNF‐α and IL‐1β, but not in a concentration‐dependent manner. Compared with other concentrations, 100 μmol/L EGCG had the most significant inhibitory effect on TNF‐α (Figure 2C,D). In regard to the inhibition of IL‐1β, there was no significant difference between the 100 μmol/L group and the 10 μmol/L group (Figure 2C). LPS promoted the production of ROS. The expression of ROS was inhibited in the 10, 20, 50, 100 μmol/L EGCG groups—especially in the 100 μmol/L group—and there was a significant difference relative to the control group (Figure 2E).

### 3.3 Effects of EGCG on Keap1/Nrf2/HO‐1 and *HDAC6* mRNA Expression in hPDLCs

**Figure 3:**
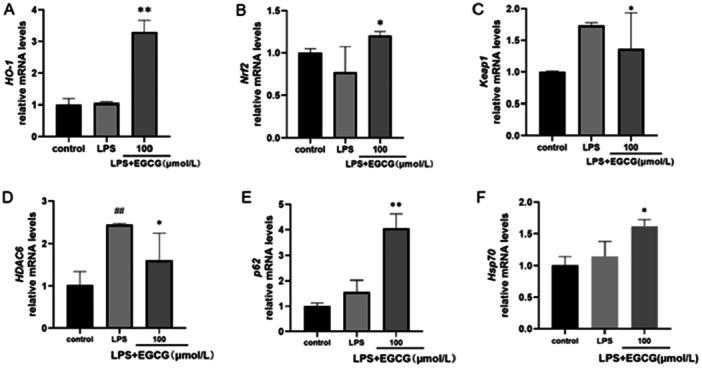
RT‐qPCR showed that EGCG could significantly promote the expression of HO‐1 and Nrf2 in hPDLCs (A and B) and inhibit the expression of Keap1 (C). EGCG inhibits HDAC6 expression; (D) promotes the expression of p62, which regulates the HDAC6 ubiquitination protease system (E); and promotes Hsp70 expression, which is regulated by HDAC6 (F). *Compared with the LPS group, *p* < 0.05; **compared with the LPS group, *p* < 0.01; ^##^compared with the control group, *p* < 0.01.

Compared with that in the control group and LPS group, the expression of HO‐1 in the EGCG group was upregulated; EGCG significantly promoted the expression of HO‐1 (Figure 3A). Compared with that in the LPS group, the expression of Nrf2 in the EGCG group was upregulated; EGCG promoted the expression of Nrf2 and inhibited the expression of Keap1 (Figure 3B,C). Compared with the control group, LPS promoted the expression of HDAC6, while EGCG inhibited the expression of HDAC6 induced by LPS (Figure 3D). The expression of p62 in the EGCG group was upregulated compared with that in the control group and LPS group (Figure 3E), while the expression of Hsp70 in the EGCG group was upregulated compared with that in the LPS group (Figure 3F). In summary, EGCG promoted the expression of the Keap1/Nrf2/HO‐1 pathway, inhibited the expression of HDAC6, and promoted the expression of p62 and Hsp70.

### 3.4 Effect of EGCG on Nrf2, NLRP3, and HDAC6 Protein Expression in hPDLCs

**Figure 4:**
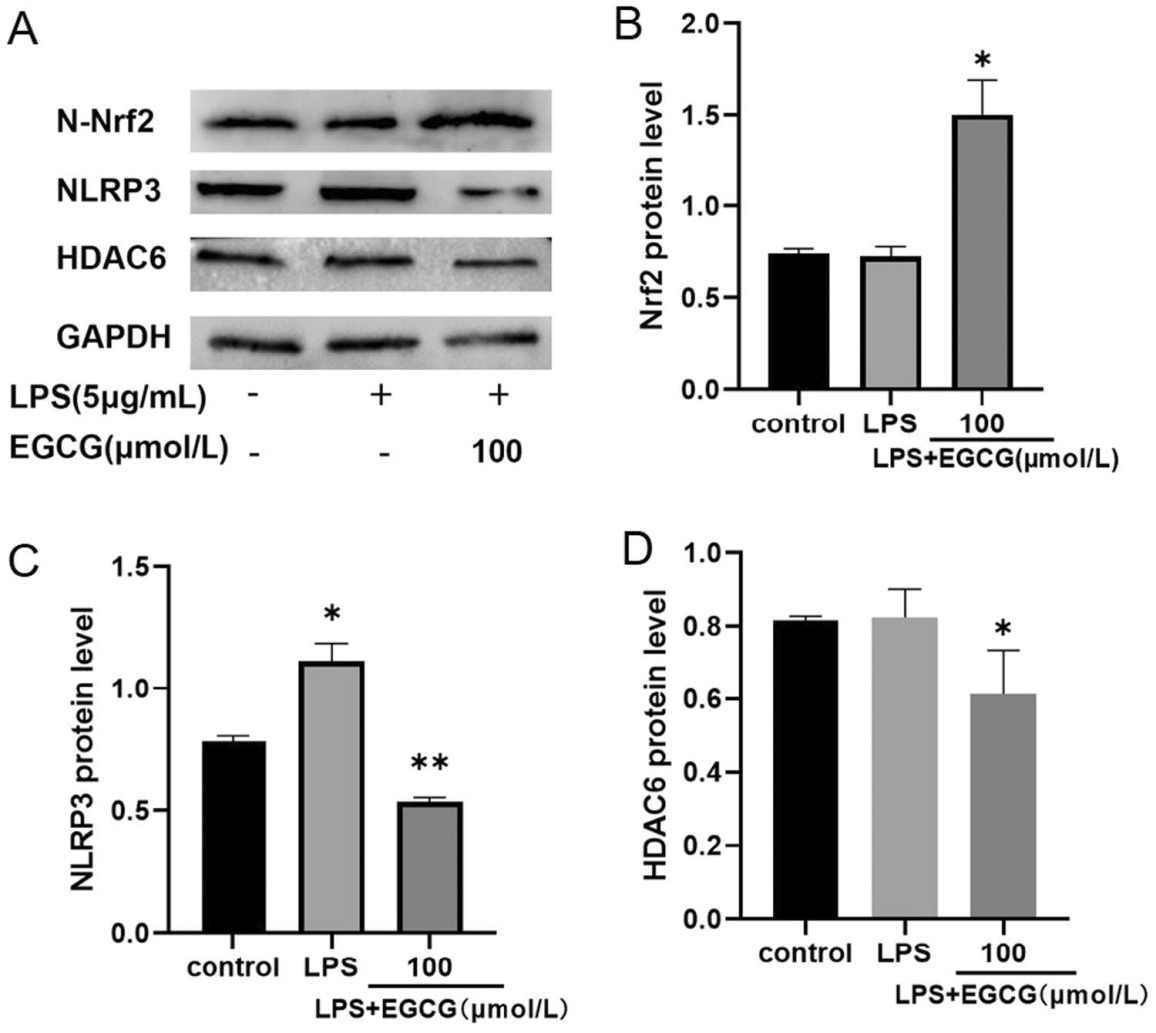
EGCG significantly promoted Nrf2 nuclear translocation in hPDLCs (A and B) and LPS‐induced NLRP3 expression was inhibited (C). EGCG also inhibits HDAC6 protein expression (D). **p < 0.01, *p < 0.05 vs. LPS group.

HDAC6 is not only involved in the expression of NLRP3, but also in the regulation of Nrf2; therefore, its role in inflammatory responses and oxidative stress is crucial. To study the anti‐inflammatory and antioxidative effects of EGCG on hPDLCs, we performed western blot experiments; results showed that Nrf2 expression in the cell nuclei of the EGCG group was upregulated, and the NLRP3 expression was increased under the action of LPS (Figure 4A–C). After EGCG treatment, NLRP3 expression was significantly inhibited (Figure 4C). Furthermore, the expression of HDAC6 was inhibited after EGCG treatment (Figure 4A,D).

## 4 Discussion

LPS and other virulence factors secreted by periodontal pathogens destroy periodontal tissue, driving the occurrence and development of periodontitis. hPDLCs, the most abundant cells in the periodontal ligament, possess the ability to produce collagen and play an important role in supporting the morphological structure of the periodontal ligament [12]. When LPS stimulates cells, it binds to Toll‐like receptors, activates inflammatory signaling pathways, and upregulates the expression of pro‐inflammatory factors such as TNF‐α, IL‐1β, and oxidative stress factors [13], resulting in periodontal tissue destruction. In this study, LPS was used to induce hPDLCs to secrete TNF‐α, IL‐1β, and ROS to establish an effective inflammatory model.

Green tea—one of the most popular drinks worldwide—is rich in polyphenols, of which EGCG is the most abundant and has significant anti‐inflammatory and antioxidant activities [14]. Studies have shown that EGCG exerts an anti‐inflammatory effect by downregulating mitogen‐activated protein kinase and NF‐κB in dental pulp fibroblasts through blocking the ligand‐stimulated TLR2 cell signaling pathway [15]. In the treatment of periodontitis, EGCG has shown excellent antibacterial and anti‐inflammatory effects and inhibition of alveolar bone resorption [16]. However, there are few studies on the role of EGCG in oxidative stress in periodontitis, and its specific mechanism of action is still unclear. Nrf2 is an inducible transcription factor that can activate a series of antioxidant proteins and Phase II detoxifying enzymes, and is a central regulator of the cellular oxidative stress response [17]. To investigate the anti‐inflammatory and antioxidative stress effect of EGCG on periodontitis, we established a rat model of periodontitis and found that EGCG could inhibit periodontitis and promote the expression of Nrf2 in this model [18]. On this basis, hPDLCs were cultured with various concentrations of EGCG for different durations to observe the effect of EGCG on hPDLC proliferation. Results showed that EGCG inhibited cell proliferation at 6, 12, and 24 h when the concentration of EGCG was 200 μmol/L. However, at a concentration of 5–100 μmol/L, EGCG had no inhibitory effect on cell proliferation. Some studies have shown that, at a concentration of higher than 100 μmol/L, EGCG causes damage to cells [19]; these findings are consistent with the results of the present experiment. It was also found that EGCG ≤ 100 μmol/L could inhibit the LPS‐induced secretion of TNF‐α, IL‐1β, and ROS in hPDLCs, with the most obvious inhibitory effect observed for 100 μmol/L. The results of RT‐qPCR showed that EGCG inhibited the expression of Keap1 and promoted the expression of Nrf2 and HO‐1, indicating that EGCG could play an antioxidative stress role in periodontitis through the Keap1/Nrf2/HO‐1 pathway. This study further demonstrates the anti‐inflammatory and antioxidative stress effects of EGCG on periodontitis at the cellular level.

Chronic periodontitis is an inflammatory disease, whose pathogenic factors include not only those related to immune pathways but also genetic factors [20]. Congenital or acquired immune deficiency, diabetes, obesity, smoking, stress, and other factors may cause epigenetic modification and destroy the host‐microbial balance. Therefore, the incidence of severe periodontitis is more significant in some populations and races [3]. In chronic inflammatory diseases, epigenetic changes may affect immune function and the inflammatory response [21]. HDAC6, a member of the HDAC family of histone deacetylases, is a cytoplasmic deacetylase with a unique structure and function. It has been shown that HDAC6‐deficient mice are protected from LPS‐induced septic shock [22, 23]. The relationship between HDAC6 and inflammatory diseases may be due to the direct participation of HDAC6 in NLRP3 activation through the ubiquitin protease system, promoting aggregate formation and autophagic degradation. Meanwhile, HDAC6 inhibitors and HDAC6‐knockout have been shown to abrogate the assembly and activation of these inflammatory bodies and alleviate inflammation [4]. Related studies on cardiac function have found that hypoxia can lead to myocardial dysfunction through epigenetic mechanisms [24]. Animal experiments have shown that the level of Nrf2/HO1 in mice with brain injury with HDAC6 silencing was significantly higher than that in the control group [25]. Therefore, HDAC6 is closely related to Nrf2 and may be an important factor in regulating this antioxidant pathway in vivo. Extant studies on the role of HDAC6 in periodontitis have found that overexpression of HDAC6 aggravates the role of LPS in promoting inflammation and apoptosis of periodontal ligament fibroblasts, and inhibits the differentiation of dental pulp stem cells into dentin [26, 27]. However, the inhibition of HDAC6 can promote osteogenic differentiation of periodontal stem cells and inhibit osteoclast formation [28].

In this experiment, we attempted to determine whether EGCG acts as an HDAC6 inhibitor to regulate LPS‐induced oxidative stress in hPDLCs. In doing so, we found that the LPS‐induced expression of HDAC6 and NLRP3 in hPDLCs was significantly upregulated, while the expression of Nrf2 was inhibited. EGCG can act as an HDAC6 inhibitor to downregulate the expression of HDAC6 and NLRP3 in hPDLCs and promote the nuclear translocation of Nrf2. It is suggested that EGCG plays anti‐inflammatory and antioxidative roles by simultaneously regulating the expression of HDAC6 and the Nrf2/HO‐1/NLRP3 signal axis in the present periodontitis cell model. Some studies have shown that p62 can directly regulate HDAC6 to inhibit the ubiquitin protease system and promote the degradation of damaged mitochondria and intracellular bacteria and viruses [29]. Hsp27 and Hsp70 in the heat shock protein (Hsp) family are substrate proteins regulated by HDAC6. The upregulation of Hsp70 can protect cells from intracellular damage caused by protease inhibition [30]. In this study, we found that EGCG promotes the expression of p62 and Hsp70; therefore, EGCG may regulate HDAC6, with p62 as the target, to promote the expression of Hsp70 and protect cells from inflammatory damage. Taken together, the results suggest that EGCG regulates the cellular antioxidant system by inhibiting HDAC6, affecting redox balance and ameliorating inflammation in hPDLCs.

## 5 Conclusions

To sum up, EGCG can inhibit the LPS‐induced inflammatory response in hPDLCs as well as cellular oxidative stress through the Keap1/Nrf2/HO‐1 pathway. Furthermore, EGCG can be used as an HDAC6 inhibitor to regulate the inflammatory response and oxidative stress in hPDLCs. However, determining whether EGCG regulates the Nrf2/HO‐1/NLRP3 signaling axis through HDAC6 requires HDAC6 to be silenced and overexpressed; this can be verified by further experiments. Periodontitis is a challenging health problem, with dedicated efforts to identify effective solutions underway globally. In this study, we demonstrate the role of EGCG as an HDAC6 inhibitor, providing new directions for the clinical prevention and treatment of periodontitis. In‐depth studies of the underlying mechanisms should strengthen the potential contribution of these findings in combating periodontitis.

## Author Contributions

F.Q. designed and directed the experiments. B.G.H. and L.J.G. contributed to the study design. Y.J. and L.X. performed most of the experiments and wrote the manuscript. C.B. and L.Y.Z. involved in project coordination, data analysis. P.Z.H., L.Y.H., and G.S. were involved in data interpretation, manuscript preparation, and submission of the manuscript. All authors have given final approval of this version to be published. All authors agreed to be accountable for all aspects of the work in ensuring that questions related to the accuracy or integrity of any part of the work are appropriately investigated and resolved. All authors have read and approved the final manuscript.

## Conflicts of Interest

The authors declare no conflicts of interest.

## Data Availability

The datasets used and/or analyzed during the current study available from the corresponding author on reasonable request.
